# Soluble checkpoint molecules as predictive biomarker for disease activity and long-term outcome in SLE

**DOI:** 10.3389/fimmu.2025.1685275

**Published:** 2025-09-25

**Authors:** Léa-Sophie Dreveton, Jakob Joachim Spencker, Hector Rincon-Arevalo, Arman Aue, Bilgin Osmanodja, Annika Wiedemann, Franziska Szelinski, Gerhard Krönke, Thomas Dörner, Eva Schrezenmeier, Ana-Luisa Stefanski

**Affiliations:** ^1^ Department of Rheumatology and Clinical Immunology, Charité- Universitätsmedizin Berlin, Berlin, Germany; ^2^ Deutsches Rheumaforschungszentrum (DRFZ), Berlin, Germany; ^3^ Department of Nephrology and Medical Intensive Care, Charité- Universitätsmedizin Berlin, Berlin, Germany; ^4^ Grupo de Inmunología Celular e Inmunogenética, Facultad de Medicina, Instituto de Investigaciones Médicas, Universidad de Antioquia UdeA., Medellín, Colombia; ^5^ Immanuel Krankenhaus Berlin Buch, Klinik für Rheumatologie und Klinische Immunologie, Berlin, Germany

**Keywords:** SLE, biomarker, soluble immune checkpoints, remission, activity, SLEDAI

## Abstract

**Background:**

Systemic lupus erythematosus (SLE) is a chronic autoimmune disease characterized by systemic inflammation and the involvement of multiple organ systems. The disease’s heterogeneity challenges clinical assessment, highlighting the need for personalized diagnostics and therapies. Soluble immune checkpoint molecules (sICPs) have emerged as potential biomarkers for predicting disease activity in other autoimmune diseases. However, the role of sICPs in SLE has not yet been delineated.

**Methods:**

In this study serum concentrations of soluble co-stimulatory (sCD25, sCD27, sCD40L, s4-1BB and sCD86) and co-inhibitory checkpoint molecules (sCTLA-4, sPD-1, sPD-L1, sPD-L2, sTim-3, sGal-9 and sLAG-3) were measured by bead-based assay in 235 SLE patients. sICPs were analyzed in relation to clinical data (SLEDAI, organ involvement, C3, C4, anti-dsDNA, Siglec- 1, and sustained DORIS/LLDAS remission). Analyses included Wilcoxon rank-sum tests, multivariable logistic regressions, receiver operating characteristic (ROC) analyses, cluster analyses, and correlation matrices.

**Results:**

Higher concentrations of sCD25, sTim-3, and sGal-9 were associated with active SLE disease (SLEDAI > 4). Cluster analysis identified highest concentrations in a SLE subgroup with more severe disease (median SLEDAI 10). These molecules correlated strongly with each other and were specifically elevated in patients with renal involvement and/ or anemia, but not with APS, skin, or joint manifestations. Low sCD25 levels were associated with sustained DORIS/LLDAS remission.

**Conclusion:**

This study highlights sCD25, sTim-3, and sGal-9 as biomarkers for active SLE and renal and hematologic involvement. Low sCD25 levels were associated with achieving long-term DORIS and LLDAS remission, underscoring the potential of sCD25 as a predictive and sensitive biomarker mandating further clinical validation.

## Introduction

Systemic lupus erythematosus (SLE) is a highly heterogenous chronic inflammatory autoimmune disease, characterized by multiple organ manifestations, mainly affecting women of childbearing age. SLE can result in severe organ damage with kidneys, nervous and vascular system, and especially joints and skin frequently affected (1). The disease process involves abnormal cellular activity of innate and adaptive immunity connected to elevated cytokine and autoantibody production together with immune complex formation all able to maintain chronic inflammatory reactions, ultimately leading to multiorgan damage accrual (2). Overall, current concepts consider a breakdown of immune homeostasis and loss of self-tolerance as key which results in abnormalities of T and B cell interaction within a milieu of extensive secretion of certain pro-inflammatory cytokines (i.e. type I and II interferon, IL-6, BAFF, APRIL) (3).

The main goal of SLE therapy is to achieve remission, as defined by the DORIS criteria (Definitions of Remission in SLE (4)) or at least to achieve a stable state of low disease activity (LLDAS), defined by minimal or controlled clinical activity on/off immunosuppression and minimal systemic glucocorticoid requirement (5). This therapeutic objective is crucial, as shown in the Systemic Lupus International Collaborating Clinics (SLICC) inception cohort, where both DORIS remission and LLDAS significantly reduce the accumulation of irreversible organ damage, enhance the patient’s quality of life and lower the overall disease burden (6). However, despite advances in diagnosis and therapy, SLE remains a heterogeneous disease with highly variable disease courses and therapeutic responses. This diversity not only complicates the clinical assessment of disease activity, but also presents a challenge and necessity for the development of personalized treatment approaches.

Given the wide variability in symptoms and disease progression together with the rising prevalence of SLE (7), early diagnosis and the systematic tracking of disease activity and progression through immunological biomarkers are becoming increasingly important to enable better disease management, individualized therapy and improve patients’ quality of life (8). Soluble immune checkpoint molecules (sICPs) were recently identified as biomarkers associated with the development, prognosis, and treatment of autoimmune disease like rheumatoid arthritis (RA) (9), systemic sclerosis (sSc) (10, 11) and (ANCA)-associated vasculitis (AAV) (12–14). Immune checkpoints represent a group of co-stimulatory and co-inhibitory molecules, which control interactions between B and T cells during an inflammatory response and play a pivotal role in the initiation, maintenance and duration of inflammatory processes triggered by (auto)immune antigens. Immune checkpoint inhibitors are known to induce immune-related adverse events (IRAEs), which mimic autoimmune diseases (15), suggesting a dysregulated immune balance as part of autoimmune pathogenesis (16–18). Moreover, agonistic modulation of inhibitory checkpoint molecules such as CTLA-4 and PD-1 has already shown efficacy in certain autoimmune diseases and is currently under investigation, highlighting their potential as promising therapeutic targets (19–21).

The aim of this study is to assess the contribution of soluble co-inhibitory and co-stimulatory checkpoint molecules in SLE and to identify potential predictive biomarkers for disease activity, specific organ manifestations and treatment outcomes in SLE patients.

## Methods

### Study design and data collection

We performed a retrospective study including all patients with a confirmed diagnosis of systemic lupus erythematosus (SLE), according to the classification criteria of EULAR/ACR 20 (8). The patients presented at the Department for Rheumatology and Clinical Immunology or the Department of Nephrology at Charité Universitätsmedizin Berlin between January 2016 and February 2023. Serum samples were collected from all patients and stored after obtaining written informed consent (EA1/002/16, EA1/302/16), EA1/009/17, EA1/215/18). A total of 235 patients and 416 patient visits were identified. Clinical data and individual blood values were extracted from medical records and electronic entries. Clinical data included disease activity via SLEDAI (Systemic Lupus Erythematosus Disease Activity Index) (22) and specific organ involvement. Laboratory values included autoantibody profile, complement factors C3, C4, serologic and urine findings and Siglec-1 expression on monocytes (a surrogate maker for the type I interferon signature (23)). To provide a comprehensive overview of the collected data, a detailed summary of all data recorded of the cohort is listed in **Supplementary Table 1**. Biobank samples per patient are summarized in **Supplementary Table 2**.

### Analyte detection

Serum concentrations of soluble co-stimulatory sCD25, sCD27, sCD40L, s4-1BB and sCD86 and co-inhibitory checkpoint molecules sCTLA-4 (soluble Cytotoxic T-Lymphocyte Activation Gene-3), sPD-1 (soluble Programmed Cell Death Protein 1), sPD-L1 (soluble programmed Death-Ligand 1), sPD-L2 (soluble programmed Death-Ligand 2), sTim-3 (soluble T-cell Immunoglobulin and Mucine-domain containing-3), sGal-9 (soluble Galectin-9) and sLAG-3 (soluble Lymphocyte Activation Gene-3) were determined using bead-based LEGENDplex multiplex analysis (BioLegend). Type I interferon (IFN) proteins IFN-α, IFN-ß, IFN-λ1 and IFN-λ2/3, as well as sAPRIL (soluble A Proliferation-Inducing Ligand) and BAFF (soluble B-cell activation factor), known cytokines involved into the pathophysiology of SLE (3, 24), were also assessed with the same bead-based method. All tests were conducted in accordance with the manufacturer´s protocol (25).

Measurements were performed on the BD FACSCanto (BD Biosciences). The results were analyzed using FlowJo software 10.7.1 (TreeStar) and Prism GraphPad Prism Version 9 (GraphPad software).

### Statistical analysis

For 235 participants, 416 blood samples were available. Descriptive statistics were applied to describe the sample regarding unique clinical characteristics of each participant (time since diagnosis, remission status, kidney involvement, associated antiphospholipid antibody syndrome) and at each time point when samples were obtained (e.g., active disease defined as SLEDAI > 4, anemia, skin involvement, articular involvement). These characteristics served as the basis for further analysis as grouping discriminants. Wilcoxon-rank-sum-test was applied to investigate the levels of sICPs between patient subgroups. For non-time-dependent variables, one representative time point per patient was included. For time-dependent variables, all available visits were used. This pragmatic approach was chosen to maximize data use while avoiding artificial inflation of patient numbers for fixed variables. More complex longitudinal models such as generalized estimating equations (GEE) were not applied, as our primary objective was to identify cross-sectional associations between biomarker levels and clinical phenotypes. The following clinical considerations were applied: for kidney involvement, the sample closest to the time of maximum creatinine was chosen; for remission and APS, the first recorded time point was used. For disease activity, anemia, skin involvement, and articular involvement, all recorded values were included in the analysis. Subsequently, multivariable logistic regression models were performed for each sICP showing significant differences in the location test. Thereby, the respective discriminant factor was set as dependent variable. To account for potential confounding, age, sex, and current daily prednisolone dose were included as covariates in all multivariable logistic regression models. If a multivariable model shows a significant fit, a univariate logistic regression model was performed with the respective sICPs as independent variable. The Receiver-Operating-Characteristic (ROC) was plotted and the area under the curve (AUC) calculated.

Besides, an unsupervised cluster analysis of certain sICPs with significant higher levels in the group of samples with a SLEDAI > 4 was conducted. Established biomarkers for SLE-Activity (anti-dsDNA, C3, C4 and SIGLEC-1) were also included. The k-mean method was chosen to investigate the possible deviating tendency of sICPs compared to established biomarkers.

Spearman coefficients were calculated between selected sICPs and the following biomarkers/clinical factors: SLEDAI; anti-dsDNA; proteinuria; creatinine; hemoglobin concentration (Hb); complement factor 3 (C3); complement factor 4 (C4); and CD163/SIGLEC-1 expressed on peripheral CD14+ monocytes. All statistical analyses were performed by using R 4.4.2 (R Foundation for Statistical Computing), utilizing packages including *dplyr* (v1.1.4), *gtsummary* (v2.2.0), *broom* (v1.0.8), *pROC* (V1.18.5), *openxlsx* (v4.2.8), *broom.helpers* (v1.20.0), *ggplot2* (v3.5.1), *tibble* (v3.2.1), *readxl* (v1.4.5), *purrr* (v1.0.2), *tidyr* (v1.3.1), *stringr* (v1.5.1), f*orcats* (v1.0.0), *scales* (v1.3.0), *RColorBrewer* (v1.1-3), *corrplot* (v0.95), *pheatmap* (v1.0.12), *survival* (v3.7-0), *stats* (v4.4.2), *utils* (v4.4.2) (26). A final spelling check was performed using ChatGPT (OpenAI).

## Results

### Cohort characteristics

According to the EULAR/ACR classification criteria 2019, we identified 235 SLE patients being followed at Charité Universitätsmedizin Berlin during 416 time points between January 2016 and February 2023. As typical for SLE, the majority of the patients were women (90%) and the median age was 38 years. The mean disease duration was 8 years and varied among patients: 17% had been diagnosed within the past 3 years, 42% between 3 to 10 years, and (41%) diagnosed over 10 years ago. Taking this into account, patients were retrospectively classified based on available longitudinal data regarding long-term remission according to DORIS and LLDAS criteria (4, 5). A total of 98 patients (41%) achieved LLDAS (mean disease duration of 13 years), of whom 60 patients (25%) (mean disease duration of 13 years) also met the criteria for DORIS remission. In contrast, 137 patients (57%) showed a rather remission-relapsing phenotype and did not achieve either DORIS or LLDAS long-term remission (mean disease duration of 11 years).

Regarding organ manifestations, kidney involvement was present in 38% of patients, with a small fraction experiencing kidney failure in our observation period (0.5%). Anemia was observed in 33% of patients, followed by the presence of an antiphospholipid syndrome (APS, 23%), skin (12%) and articular manifestations (6%). The SLE patients in our cohort received individual treatment regimes, so medication changed over time according to their disease activity. At the first presentation in our clinic during the observational period, most of the patients received hydroxychloroquine (58%) and prednisolone (45%, mean dose per day was 4,8 mg/d) followed by azathioprine (23%) and mycophenolate mofetil (14%) as well as initial biologics (belimumab in 5%, rituximab in 6%). Demographic and clinical characteristics as well as treatment regimen for all study participants are summarized in **Table 1**.

**Table 1:** Demographic and clinical characteristics.

Characteristic		N = 235
Age (Median / IQR)		38 (30,68; 52,74)
Sex	f	211 (90%)
	m	24 (10%)
Time since diagnosis	< 3 years	40 (17%)
	3-10 years	98 (42%)
	> 10 years	97 (41%)
Sustained DORIS remission*	yes	60 (25%)
	no	175 (73%)
Sustained LLDAS remission^#^	yes	98 (42%)
	no	137 (58%)
Kidney involvement		90 (38%)
Anemia		78 (33%)
Skin involvement		29 (12%)
Articular involvement		15 (6%)
Antiphospholipidsyndrom		54 (23%)
Medication	hydroxychloroquine prednisolone mean dose per day (mg/D) (IQR) azathioprine mycophenolate mofetil belimumab rituximab	137 (58%) 106 (45%) 4,8 (0; 5) 53 (23%) 33 (14%) 12 (5%) 15 (6%)

*Defined as cSLEDAI 0, ≤  5 mg/day Prednisolone, and no flares during the observation period.

^#^Defined as SLEDAI ≤  4, ≤  7.5 mg/day Prednisolone, and a maximum of one flare during the observation period.

### Higher levels of sCD25, sTim-3 and sGal-9 are characteristic of active SLE

In a first step, we wanted to identify possible biomarkers for SLE activity. Therefore, the study participants were categorized at each time point of their presentation in our clinic as either inactive or active according to a SLEDAI score above 4. All recorded time points were included in this analysis. First, we performed a Wilcoxon rank sum test using the median values of checkpoint molecules and identified significant higher levels of sCD25, sCTLA-4, sGal-9, sLAG-3, sPD-1, sPD-L1, sTim-3, and sCD27 in active compared to inactive patients (**Supplementary Table 3**).

**Figure 1:**
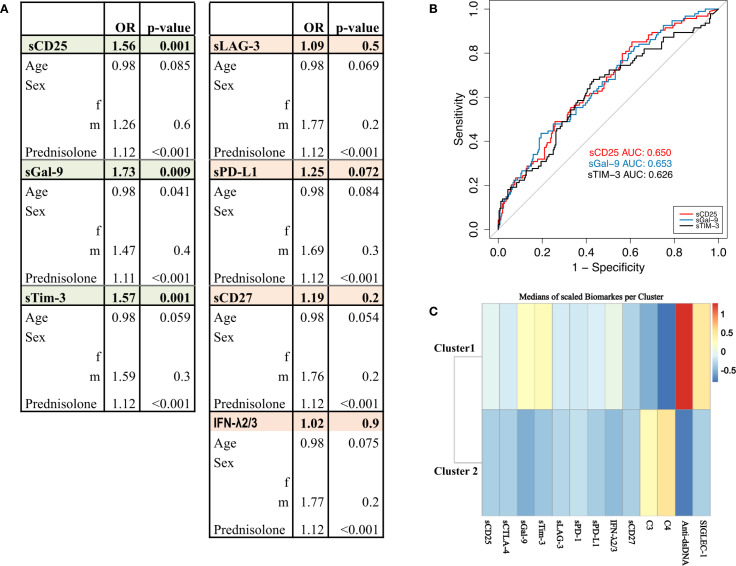
SLEDAI. **(A)** Circulating levels of sCD25, sTim-3 and sGal-9 are higher in the disease group (SLEDAI > 4) compared with patients in remission or LLDAS (SLEDAI ≤ 4). Multivariable logistic regression model adjusted for age, sex, and prednisolone dose. Significant results (p<0.05) are highlighted in green, non-significant results are shown in orange. **(B)** ROC curves displaying the predictive performance of univariate logistic regression models for biomarkers sCD25 (red), sTim-3 (black), and sGal-9 (blue) differentiate SLEDAI active vs. inactive states. **(C)** Cluster analysis of biomarker levels identifies two groups based on similarities in biomarker expression. The analysis represents the median values of biomarkers per cluster, with higher biomarker levels shown in red and lower levels in blue. Highlighting patterns of biomarker distribution across the cluster.

To rule out possible confounders and to evaluate the odds of disease activity in relation to the levels of these checkpoint molecules, we conducted a multivariable logistic regression analysis. We defined prednisolone dose, age and sex as possible confounder and adjusted accordingly. Interestingly, higher concentrations of sCD25, sTim-3 and sGal-9 were associated with active disease (**Figure 1A**), confirming these three molecules as the most robust ones related to higher lupus activity. The prednisolone dose appeared to be the most significant confounder when comparing active versus inactive patients in our system, as it is administered in higher doses or as a pulse therapy in active patients. We assessed the predictive performance of these molecules for active SLE using univariate logistic regression and ROC analysis. Interestingly, the ROC curve for sCD25 demonstrated an AUC value of 0.650, sGal-9 0.653 and sTim-3 an AUC value of 0.626 (**Figure 1B**).

### Cluster analysis

To identify potential subgroups of patients with similar biomarker profiles, we conducted an unsupervised cluster analysis of all patients with active SLE (SLEDAI > 4) including classical biomarkers, such as anti-dsDNA titer, complement C3, C4 levels and CD169/Siglec-1 expression related to type I IFN as well as the most identified sICPs (sCD25, sGal-9, sTim-3, sPD-L1, IFN-λ2/3 and sCD27) (**Figure 1C**). The resulting plot illustrates the mean values of the biomarkers within the different clusters. Two main clusters were identified: cluster 1 containing 26 patients and cluster 2 containing 39 patients. Detailed characteristics of the patients in each cluster are given in **Supplementary Table 4**.

Cluster 1 included SLE patients with significantly higher disease activity (median SLEDAI 10) compared to Cluster 2 (median SLEDAI 6). In addition to higher anti-dsDNA titers and hypocomplementemia, patients in cluster 1 exhibited also significantly higher levels of Siglec-1, sCD25, sTim-3, and sGal-9 compared to those in Cluster 2, along with higher levels of IFN-λ2/3. The clustering indicates that within the active SLE cohort, there is a subgroup with known conventional lupus activity markers as well as elevated checkpoint molecule levels. This pattern suggests a subgroup with high disease activity and elevated checkpoint molecules, potentially linked to more severe manifestations.

### Soluble checkpoint molecules as biomarkers for renal and hematologic SLE manifestations

**Figure 2:**
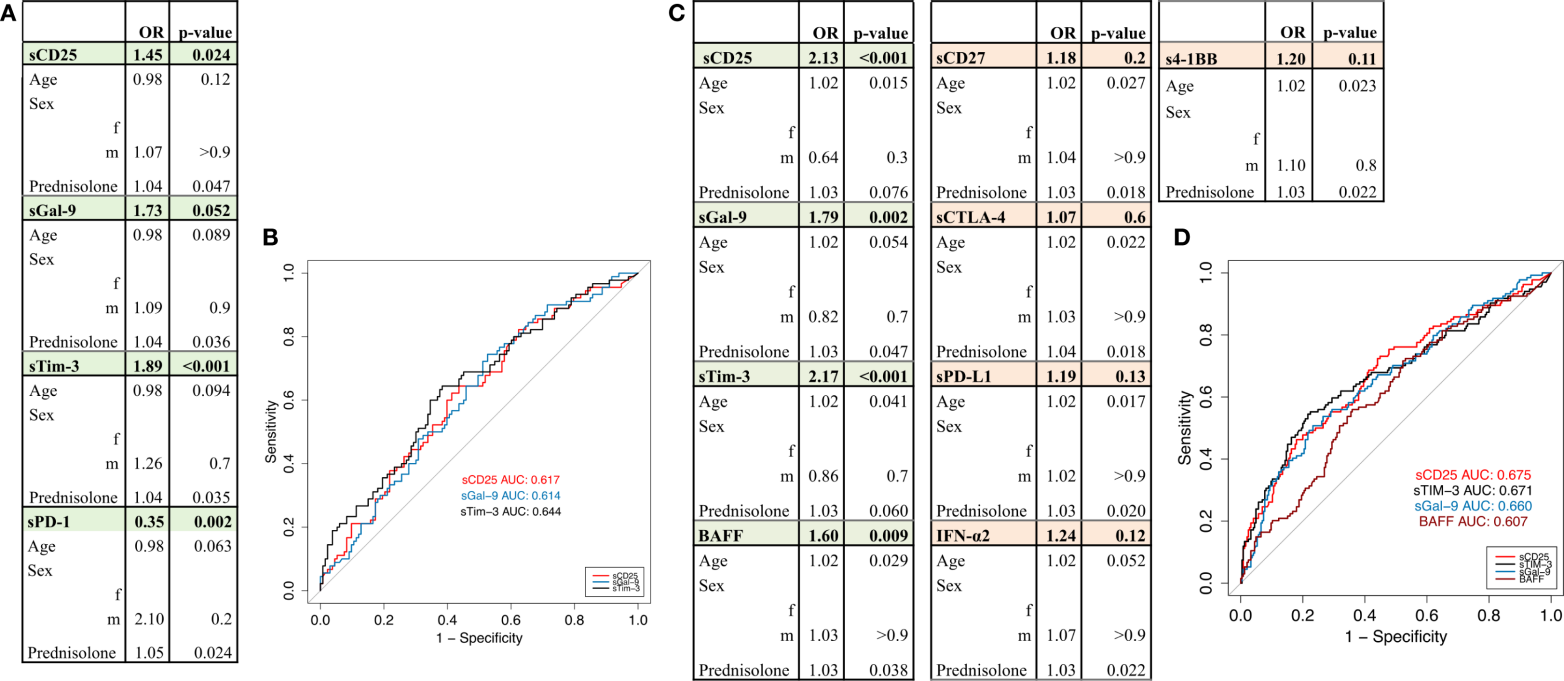
Kidney involvement and anemia. **(A)** Circulating levels of sCD25, sTim-3, sGal-9 and sPD-1 are higher in patients with renal involvement compared to patients without renal involvement. Multivariable logistic regression model adjusted for age, sex, and prednisolone dose. Significant results (p<0.05) are highlighted in green, non-significant results are shown in orange. **(B)** ROC curves displaying the predictive performance of univariate logistic regression models for biomarkers sCD25 (red), sTim-3 (black), and sGal-9 (blue) in differentiating patients with and without renal involvement. **(C)** Circulating levels of sCD25, sTim-3, sGal-9 and BAFF are higher in patients with anemia compared to patients without anemia. Multivariable logistic regression model adjusted for age, sex, and prednisolone dose. Significant results (p< 0.05) are highlighted in green, non-significant results are shown in orange. **(D)** ROC curve displaying the predictive performance of univariate logistic regression models for sCD25 (red), sTim-3 (black), sGal-9 (blue) and BAFF (brown) in differentiating patients with versus without anemia.

Given the heterogeneity of the disease, we aimed to identify in a next step, the role of sICPs in specific organ manifestations of SLE. According to the cohort characteristics, we focused on renal, hematologic (anemia), skin and articular manifestations as well as the presence of an associated APS. Therefore, we compared the group of patients with increased disease activity in one specific organ system with patients who never experienced activity in this organ system, respectively. Using Wilcoxon rank sum tests, we observed significantly higher levels of sCD25, sTim-3, sGal-9 and sPD-1 in patients with renal involvement (**Supplementary Table 5**). To account for potential confounders such as age, sex, prednisolone dose, we performed multivariable logistic regression and ROC curve analysis. This evaluation revealed sCD25, sTim-3 and sGal-9 as the most robust soluble checkpoint molecules related to renal manifestations (**Figures 2A, B**, **Supplementary Figure 1**). In patients presenting with anemia, Wilcoxon rank sum tests also revealed elevated serum concentrations of 4-1BB, sCD25, sCTLA-4, sGal-9, sPD-L1, sTim-3, IFN-α2, sCD27 and BAFF compared to patients without anemia (**Supplementary Table 6**). Multivariable logistic regression and ROC curve analysis confirmed these associations only for sCD25, sGal-9, sTim-3 and BAFF (**Figures 2C, D**). A detailed analysis of the patient distribution showed that 38% of the patients with kidney involvement also presented concomitantly with anemia.

Differences in sICPs levels were observed in patients with skin manifestation in univariable analysis for IFN-λ2/3 (**Supplementary Table 7**), but not in the multivariable model (**Supplementary Table 8**). Interestingly, no significant differences of sICPs level were found in patients with skin (**Supplementary Tables 7**, **8**) or articular manifestations (**Supplementary Table 9**) or associated APS (**Supplementary Table 10**), respectively.

**Figure 3:**
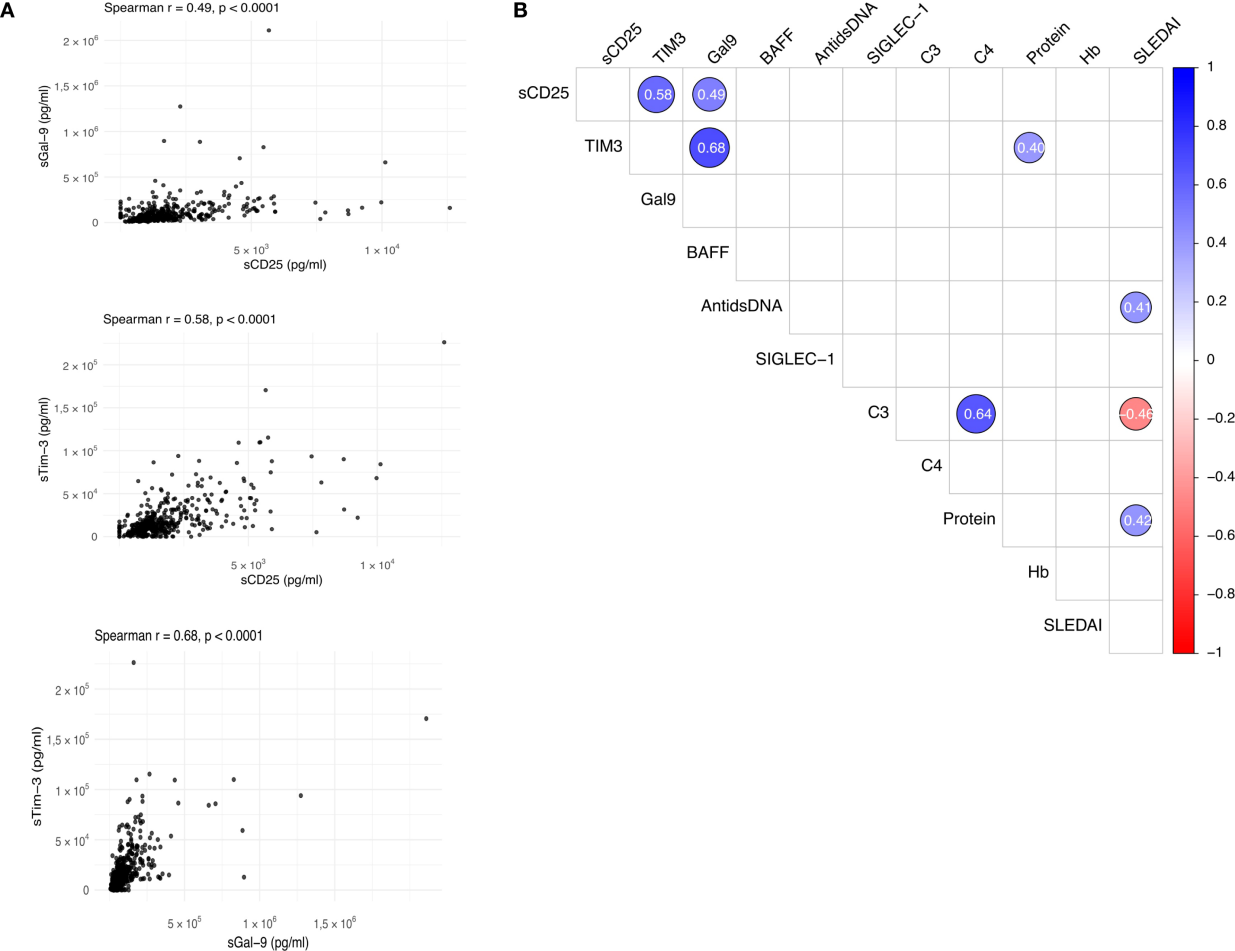
Correlation. **(A)** Scatter plots showing correlations between sCD25, sTim-3 and sGal-9. Pearson correlation coefficients (r) and p-values are provided for each comparison. **(B)** Correlation matrix displaying associations between biomarkers (sCD25, sTim-3 and sGal-9 and BAFF) and other parameters (anti-dsDNA, SIGLEC-1, C3, C4 Proteinuria, Hb, SLEDAI and cSLEDAI). Correlation coefficients are represented by color intensity, with blue indicating positive and red negative correlations.

To evaluate the relationship between the checkpoint molecules and clinical parameters, we performed a correlation matrix (**Figures 3A, B**): strong positive correlations between sCD25, sTim-3, and sGal-9 were found suggesting that they are likely involved in interrelated pathophysiologic mechanisms driving their upregulation and appearance in the circulation. With respect to clinical data, a direct correlation was seen between sTim-3 level and proteinuria. Notably, no correlation was observed between the significant sICPs and Siglec-1 expression on peripheral CD14+ monocytes suggesting that these molecules are probably independent of type I IFN upregulation.

### Low sCD25 levels as robust biomarkers for sustained remission.

To assess long-term remission in patients with SLE, we evaluated their status based on established criteria for DORIS and LLDAS during the observational period. A total of 98 out of 235 patients (42%) met the criteria for LLDAS, of whom 60 patients (25%) also fulfilled the criteria for DORIS remission.

**Figure 4:**
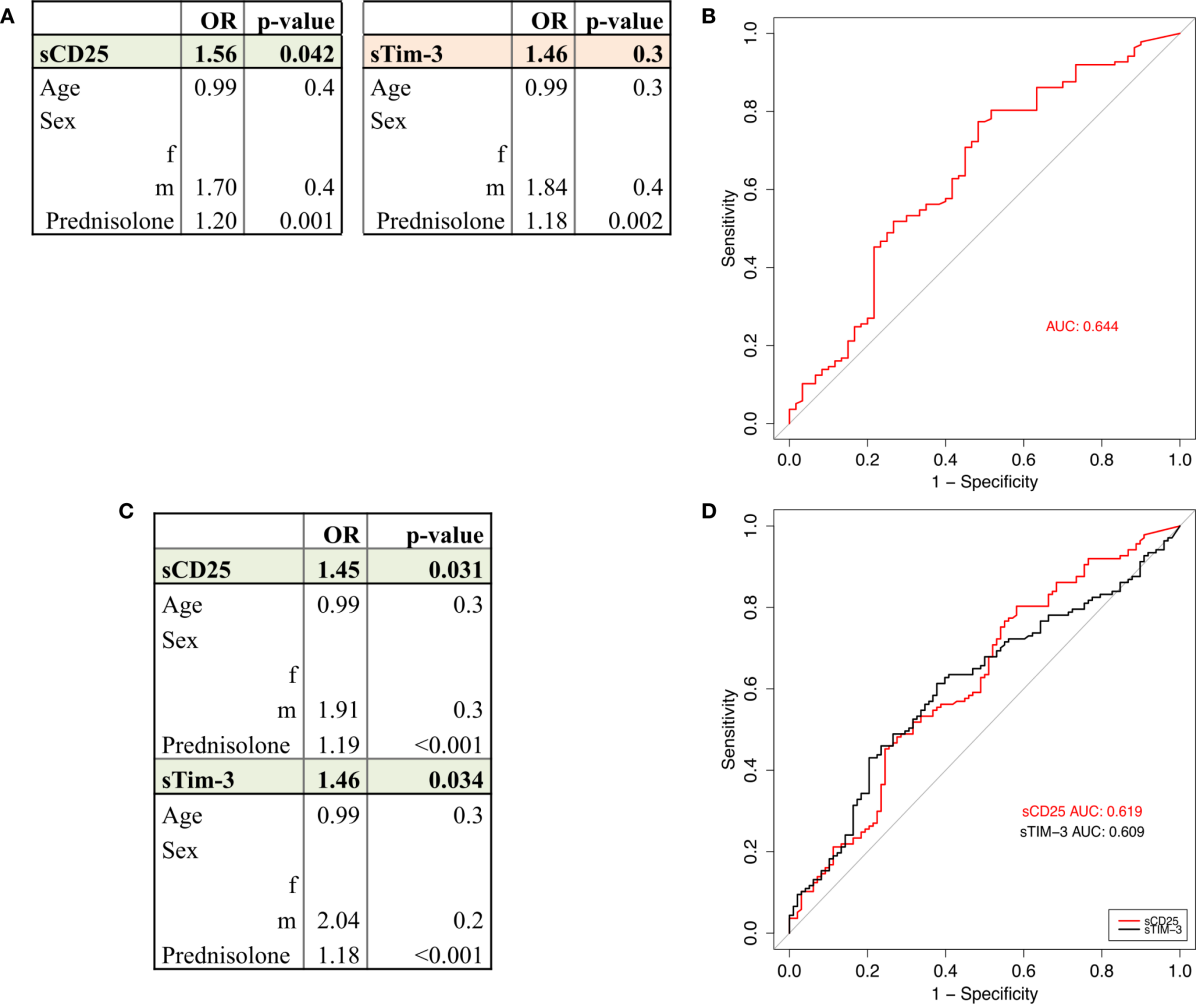
DORIS remission and LLDAS remission. **(A)** Circulating levels of sCD25 and sTim-3 are lower in patients in DORIS Remission compared to those neither in DORIS nor in LLDAS. Multivariable logistic regression model adjusted for age, sex, and prednisolone dose. Significant results (p<0.05) of sCD25 are highlighted in green, non-significant results (sTIM-3) are shown in orange. **(B)** ROC curves displaying the predictive performance of univariate logistic regression models for biomarker sCD25 (red). **(C)** Circulating levels of sCD25 and sTim-3 are lower in patients in LLDAS remission compared to those not in LLDAS. Multivariable logistic regression model adjusted for age, sex, and prednisolone dose. Significant results (p< 0.05) are highlighted in green, non-significant results are shown in orange. **(D)** ROC curve displaying the predictive performance of univariate logistic regression models for sCD25 (red) and sTim-3 (black).

Patients were retrospectively classified into four distinct groups: those achieving DORIS remission, those achieving LLDAS remission, those achieving LLDAS remission but not DORIS, and those who did not achieve either DORIS or LLDAS remission. In an initial step, Wilcoxon rank sum tests revealed that patients in sustained DORIS or LLDAS remission displayed significantly lower levels of sCD25 and sTim-3 (**Supplementary Tables 11**, **13**, **14**). Multivariable logistic regression (**Supplementary Table 12**, **Figure 4**) revealed that lower concentrations of sCD25 were consistently associated with a higher likelihood of achieving both DORIS and LLAS remission. In contrast, patients who did not achieve either DORIS or LLDAS remission exhibited significantly elevated levels of sCD25 and sTim-3. (**Supplementary Table 11**, **Figures 4A, C**).

The prednisolone dose appeared to be the most influential confounder in comparing remission and non-remission groups, as it is administered in higher doses or as pulse therapy in patients with active disease who fail to achieve DORIS or LLDAS remission. (**Supplementary Table 11**, **Figures 4A, B**). None of the investigated molecules could differentiate between patients in DORIS remission and patients fulfilling LLDAS criteria without DORIS remission.

To further evaluate the predictive power of sCD25 and sTim-3, univariate models were calculated, and receiver operating characteristics (ROC) analyses were performed. The predictive performance of sCD25 was particularly strong in the DORIS vs. neither DORIS nor LLDAS comparison (AUC=0.619) (**Figure 4B**), while sTim-3 added predictive values in the LLDAS vs. non-LLDAS comparison (AUC=0.609) (**Figure 4D**).

## Discussion

In this study, we investigated the role of various immune checkpoint molecules in relation to disease activity, specific organ manifestations and long-term outcome in patients with SLE. We included molecules belonging to different co-stimulatory and co-inhibitory pathways, to be able to assess the inflammatory orchestration related to their immunobiology mechanisms. To understand dynamic changes during disease progression, we analyzed data at multiple time points to identify potential temporal dynamics in SLE.

As key findings, higher serum concentration of the co-stimulatory molecule sCD25 as well as of the co-inhibitory molecules, sTim-3 and sGal-9 were characteristic in active SLE patients, specifically in patients with kidney involvement and/or anemia. However, although skin and joint manifestations are components of SLEDAI, organ specific analyses did not reveal independent associations with cutaneous and articular involvement or presence of APS, supporting the hypothesis that specific sICPs may play different roles in certain organ manifestations. Moreover, these molecules together with the conventional activity markers (anti-ds-DNA titers and hypocomplementemia) identified a more severe disease phenotype, consistent with our unsupervised cluster analysis. sCD25 was uniquely associated with sustained DORIS/LLDAS remission, indicating its potential role as a biomarker for identifying patients eligible for treatment de-escalation and reduced pharmacologic exposure.

Although membrane-bound immune checkpoint molecules are well characterized, the origin and function of their circulating soluble forms remains incompletely understood. In addition to proteolytic cleavage by metalloproteases and/or alternative pre-mRNA splicing (27), they may also arise from passive release during enhanced lymphocyte death or fragility, a phenomenon observed in SLE (28). In our cohort, however, leukocyte counts did not correlate with sCD25, sTim-3, or sGal-9 (**Supplementary Figure 3**), supporting shedding and/or splicing as the more likely predominant sources.

sCD25 also known as sIL-2R (soluble interleukin 2 receptor), is a key co-stimulatory receptor for the human immune system, playing a crucial role for an adequate T cell response (via proliferation of effector T cells) as well as in immune tolerance and regulation of CD4+ T lymphocytes (via T regulatory cells/Treg (29),). Elevated sCD25 values are known to be present in patients with granulomatous lesions and are included in the classification criteria for sarcoidosis (30) and hemophagocytic lymphohistiocytosis (HLH) (31). In SLE, acquired interleukin-2 (IL-2) deficiency disrupts the balance between Treg and pathogenic effector/memory CD4^+^ T cells, impairing Treg homeostasis and promoting autoreactive T-cell activity and chronic inflammation (32). Accordingly, low-dose IL-2 approaches have shown immunomodulatory effects in SLE, including Treg expansion in early studies (33, 34), however, clinical efficacy remains uncertain (35). In this context, higher circulating levels of sCD25 may indicate an inefficient or abnormal immune regulation, potentially compensating impaired Treg dysfunction and IL-2 deficiency. In a clinical context, elevated levels of sCD25 in SLE have been reported to be associated with higher disease activity and with lupus nephritis (36). In our cohort, we could furthermore identify low sCD25 as a key biomarker for long-term DORIS and LLDAS remission.

The next identified significant sICPs Tim-3 and Gal-9, describe a receptor-ligand interrelationship. Tim-3 is a unique inhibitory receptor, being largely restricted to terminally differentiated, inflammatory IFN-γ-producing T helper 1 (Th1) CD4+ and CD8 T cells (37). IFN-γ is a central driver of SLE pathogenesis, particularly in lupus nephritis, and remains insufficiently controlled by current therapies (38, 39). At the same time, IFN-γ regulates multiple inhibitory checkpoint molecules including Tim-3/Gal-9 and PD-1/PD-L1 pathways (40, 41). Tim-3, initially identified as a marker of IFN-γ-producing CD4+ and CD8+ T cells (40), has been reported to have multiple different ligands, whereby the interaction with galectin 9 (Gal-9) seems to reflect the main inhibitory pathway, leading to T cell exhaustion and ultimately to cell death (42). In line with this, several subsets of T and B cells in SLE exhibit signs of functional exhaustion or remain in a post-activated state (43–45), a phenotype, which may be linked to enhanced activation of inhibitory immune checkpoints (46). Soluble Tim-3 is supposed to be mainly a result of metalloproteinase-dependent cleavage that facilitates its shedding from the cell surface (47) and has functionally the ability to reduce IL-2 production by T cells (48). In this context, higher levels of sTim-3 may reflect an inefficient attempt to counterbalance the continuous immune activation in SLE, by simultaneously accentuating the IL-2 deficiency. Our study highlights the role of the Tim-3 – Gal-9 pathway in SLE and goes in line with previous work showing increased Tim-3 expression upon SLE CD3^+^CD4^+^ T cells (49) as well as higher values of sTim-3 in SLE compared with healthy controls (50). Our association of sTim-3 with SLE kidney involvement underlines the previous observation of Tim-3 expression in the renal interstitial and tubular epithelial cells from lupus nephritis patients (51). An observation of our cohort is the association of increased sTim-3 and anemia that is undergoing further delineation.

With respect to Gal-9, there is evidence that it may be induced by Tim-3 as an inhibitory feedback mechanism (37), and can also exert Tim-3-independent effects (42). Previous studies have reported significantly higher sGal-9 levels in SLE patients compared to healthy controls, including a correlation with lupus disease activity (52, 53). Our data provide additional evidence towards a relationship between Gal-9 levels with specific organ manifestations like kidney involvement and anemia. Interestingly, although sTim-3 and sGal-9 showed strong associations with active SLE, particularly with renal and hematologic manifestations, they were not linked to sustained DORIS/LLDAS remission. Further research it needed to elucidate the molecular mechanism underlying sustained remission. While sPD-1 showed a significant association with renal involvement in both univariable and multivariable logistic regression analyses, its poor discriminatory performance in ROC analysis (AUC 0.369) suggests that sPD-1 lacks clinically utility as a biomarker in this context.

BAFF, also known as BlyS (B-lymphocyte stimulator), is a cytokine belonging to the tumor necrosis factor (TNF) family and plays a key role in B-cell activation, differentiation and survival (24). In our study, BAFF was significantly associated with anemia in SLE patients. The underlying mechanism resulting in increased BAFF may comprise increased cleavage and/or production. As there is only a moderate effect of belimumab, a monoclonal antibody targeting BAFF, on anemia (54), the current finding provides the basis for further mechanistic studies.

To sum up, our findings indicate that certain checkpoint molecules are intimately associated with increased disease activity, specific clinical manifestations and able to predict long-term outcome. The strong correlations between sCD25, sTim-3, and sGal-9 levels suggest their involvement in shared immune T/B abnormalities possibly related to increased IFNγ.

The main limitation of this study is its retrospective nature including lack of standardized data collection and various patient observation periods. Additionally, treating each time point as an independent data source rather than analyzing time-course data of individual patients precluded more stringent data assessments. Individual overlap of organ manifestations may be another significant confounder of the current analyses.

Our study identified a significant diagnostic value for sCD25, sTim-3, sGal-9 and BAFF as biomarkers for subsets of SLE patients and predicting outcome. The results enhance our understanding of the role certain CPM for disease activity and candidate for innovative diagnostic and therapeutic biomarkers. Most notably, low sCD25 levels were particularly associated with long-term DORIS and LLDAS remission, highlighting its potential as a marker for sustained disease control and potentially holding promise to guide safely treatment de-escalation or discontinuation. Nevertheless, translation into clinical practice will require assay standardization, validation in independent cohorts, and longitudinal studies to confirm its relevance as a biomarker guiding personalized therapeutic strategies in SLE.

## Data Availability

The raw data supporting the conclusions of this article will be made available by the authors, without undue reservation.
