# Supplementary material for: Soluble checkpoint molecules as predictive biomarker for disease activity and long-term outcome in SLE
Source: Front Immunol. 2025 Sep 25;16:1685275. doi: 10.3389/fimmu.2025.1685275 (PMC12507860; doi:10.3389/fimmu.2025.1685275)

Supplementary Material

Soluble checkpoint molecules as predictive biomarker for disease activity and long-term outcome in SLE

Running Title: Checkpoint molecules in Systemic lupus erythematosus

Léa-Sophie Dreveton[1,2,3], Jakob Joachim Spencker[3], Hector Rincon-Arevalo[1,2,3,4], Arman Aue[2,3,5], Bilgin Osmanodja[3], Annika Wiedemann[1,2], Franziska Szelinski[1,2], Gerhard Krönke[1,2], Thomas Dörner[1,2], Eva Schrezenmeier[2,3], Ana-Luisa Stefanski[1,2]

[1] Department of Rheumatology and Clinical Immunology, Charité- Universitätsmedizin Berlin, Berlin, Germany.
[2] Deutsches Rheumaforschungszentrum (DRFZ), Berlin, Germany.
[3] Department of Nephrology and Medical Intensive Care, Charité- Universitätsmedizin Berlin, Berlin, Germany.
[4] Grupo de Inmunología Celular e Inmunogenética, Facultad de Medicina, Instituto de Investigaciones Médicas, Universidad de Antioquia UdeA, Medellín, Colombia.
[5] Immanuel Krankenhaus Berlin Buch, Klinik für Rheumatologie und klinische Immunologie

Corresponding Author:
Léa-Sophie Dreveton
Charité – Universitätsmedizin Berlin, Charitéplatz 1, 10117 Berlin
leasophie.dreveton@googlemail.com

Contents:

Supplementary Table 1. Overview of collected categories for patient data

Supplementary Table 2. Timely distribution of biobank samples per patient

Supplementary Table 3. SLEDAI > 4 (active) vs. SLEDAI ≤ 4 (inactive) [all values], medians and p-values of Wilcoxon rank-sum test

Supplementary Table 4. Characteristics grouped by cluster. Significant values (p < 0.05) are highlighted in orange.

Supplementary Table 5. Absent kidney involvement vs. kidney involvement [minimal time difference to creatinine max], medians and p-values of Wilcoxon rank-sum test. Significant differences (p-values < 0.05) are highlighted in grey.

Supplementary Table 6. Absent anemia vs. anemia [all values], medians and p-value of Wilcoxon rank-sum test. Significant differences (p < 0.05) are highlighted in grey.

Supplementary Table 7. Absence of skin involvement vs. skin involvement [all values] medians and p-value of Wilcoxon rank-sum test. Significant differences (p < 0.05) are highlighted in grey.

Supplementary Table 8. Circulating levels of IFN-λ2/3 and IL-2 are not higher in patients with skin involvement compared to patients without skin involvement. Multivariable logistic regression model adjusted for age, sex, and prednisolone dose. Non-significant results (p>0.05) are highlighted in orange.

Supplementary Table 9. Absent articular involvement vs. articular involvement [all timepoints] medians and p-values of Wilcoxon rank-sum test

Supplementary Table 10. Absent APS findings vs. APS [first timepoints] medians and p-value of Wilcoxon rank-sum test. Significant p-values (p < 0.05) are highlighted in grey.

Supplementary Table 11. Comparison between patients in DORIS remission and those not in DORIS remission [first timepoint] (including patients in LLDAS but not in DORIS remission) (medians and p-value of Wilcoxon rank-sum test are listed). Significant p-values (p < 0.05) are highlighted in grey.

Supplementary Table 12. Circulating levels of sCD25 and sTim3 are lower in patients in DORIS remission compared to those not in DORIS remission.
Multivariable logistic regression model adjusted for age, sex, and prednisolone dose. Non-significant results (p>0.05) are highlighted in orange.

Supplementary Table 13. DORIS remission versus neither DORIS nor LLDAS remission [first timepoint], medians and p-value of Wilcoxon rank-sum test. Significant p-values ($p < 0.05$) are highlighted in grey.

Supplementary Table 14. LLDAS remission versus non-LLDAS remission [first timepoint], medians and p-value of Wilcoxon rank-sum test. Significant p-values ($p < 0.05$) are highlighted in grey.

Supplementary Figure 1. ROC curve displaying the predictive performance of univariate logistic regression models for sPD-1 (brown), in differentiating patients with kidney involvement and no kidney involvement.

Supplementary Figure 2. Spearman correlation Matrix [active], samples with SLEDAI > 4). Correlation matrix displaying associations between biomarkers (sCD25, sTim-3 and sGal-9 and BAFF) and other parameters (anti-dsDNA, SIGLEC-1, C3, C4 Proteinuria, Hb, SLEDAI and cSLEDAI). Correlation coefficients are represented by color intensity, with blue indicating positive and red negative correlations.

Supplementary Figure 3. Correlations between leukocyte counts and soluble checkpoint molecules.
Scatter plots showing correlations between sCD25, sTim-3 and sGal-9 and leukocyte count. Pearson correlation coefficients (r) and p-values are provided for each comparison.

Supplementary Table 1. Overview of collected categories for patient data

**Categories**
Age
Place of treatment (ambulant or hospitalized)
Biological Sex
Diagnoses given to the patient
Year of diagnosis
CD8 cell count[1]
CD4 cell count[1]
Natural Killer (NK) cell count[1]
B cell count[1]
SIGLEC-1 level[1]
Memory cell count[1]
Plasmablast cell count[1]
Naive B cell count[1]
C-reactive protein level[2]
Complement component C3 level[2]
Complement component C4 level[2]
Hemoglobin level[2]
White blood cell count[2]
Platelet count[2]
Serum creatinine level[2]
Maximum creatinine level in our observationperiod[2]
Protein level[2]
Antinuclear antibody titer[2]
Andi-double stranded DNA[2]
Rheumatoid factor IgA[2]
Rheumatoid factor IgG[2]
Anti-cyclic citrullinated peptide[2]
Anticardiolipin antibody IgM level[2]
Anticardiolipin antiboidy IgG level[2]
Presence of blood and protein in urine[2]
Presence of articular involvement at the timepoint
Presence of kidney involvement
Presence of skin involvement at the timepoint
Low white blood cell count
Low platelet count
Class of lupus nephritis
Systemic Lupus Erythematosus Disease Activity Index (SLEDAI)
Clinical Systemic Lupus Erythematosus Disease Activity Index (cSLEDAI)
medication the patient is taking
Number of disease flares

Whether the patient is in remission, relapsing remitting or permanently active
[1]Measured in the local laboratory via FACS (fluorescence-activated cell sorting)<br>[2]Measured in the central laboratory

Supplementary Table 2. Timely distribution of biobank samples per patient

		**N = 235**
Biobank samples per patient in total	One sample	143 (61%)
	Between 1 and 4	80 (34%)
	4 and more	12 (5%)
Biobank samples per patient longitudinal	One sample or all sample within one year	171 (73%)
	3 year range	34 (15%)
	6 year range	28 (12%)
	More than 6 years	2 (0.4%)

Supplementary Table 3. SLEDAI > 4 (active) vs. SLEDAI ≤ 4 (inactive) [all values], medians and p-values of Wilcoxon rank-sum test

	**inactive (n = 252)**	**active (n = 94)**	**p-value**
s4-1BB	85 (153)	80 (117)	0.838
sCD86	300 (377)	369 (455)	0.206
sCD25	1,282 (1,1193)	1,793 (1,959)	<0.001
sCTLA-4	0 (10)	3 (15)	0.073
sGal-9	69,857 (81,919)	100,767 (123,498)	<0.001
sLAG-3	1,607 (3,108)	2,638 (3,382)	0.026
sPD-1	32 (63)	47 (75)	0.067
sPD-L1	92 (219)	153 (316)	0.011
sTim-3	12,823 (16,741)	18,124(26,392)	<0.001
IFN-α2	0 (2)	0 (4)	0.157
IFN-λ2/3	0 (18)	4 (30)	0.011
IFN-λ1	23 (77)	40 (79)	0.248
sCD27	20,723 (23,865)	27,474 (36,786)	0.007
sAPRIL	33,216 (39,098)	37,243 (37,480)	0.955
BAFF	670 (1,140)	909 (2,294)	0.142
IFN-ß	24 (70)	17 (56)	0.110
sPD-L2	8,336 (5,212)	9,805 (6,232)	0.065
sCD40L	27,411 (27,290)	25,915 (28,514)	0.746

Median in pg/ml (IQR)

Supplementary Table 4. Characteristics grouped by cluster. Significant values ($p < 0.05$) are highlighted in orange.

Characteristic	Cluster 1 N = 26	Cluster 2 N = 39	p-value
Age	34 (19)	41 (19)	0.341
Sex			0.296
f	24 (92%)	32 (82%)	
m	2 (7.7%)	7 (18%)	
Anemia			0.076
no	19 (73%)	35 (92%)	
yes	7 (27%)	3 (7.9%)	
Unknown	0	1	
Kidney			0.54
no	10 (38%)	18 (46%)	
yes	16 (62%)	21 (54%)	
SLEDAI	10.0(5.0)	6.0(4.0)	0.002
YD			0.042
<3 Jahre	10 (38%)	5 (13%)	
3-10 Jahre	8 (31%)	13 (33%)	
>10 Jahre	8 (31%)	21 (54%)	
sCD25	2,364 (3,323)	1,758 (1,129)	0.006
sCTLA-4	5 (10)	0 (11)	0.062
sGal-9	166,843 (94,860)	70,705 (61,501)	<0.001
sTim-3	38,905 (33,922)	14,442 (15,942)	<0.001
sLAG-3	2,505 (4,372)	1,983 (2,502)	0.275
sPD-1	52 (86)	36 (54)	0.201
sPD-L1	188 (404)	114 (191)	0.021
IFN-λ2/3	22 (57)	0 (13)	0.011
sCD27	27,777 (62,160)	26,484 (39,978)	0.249
C3	660 (228)	840 (290)	<0.001
C4	75 (53)	180 (110)	<0.001
Anti-dsDNA	201 (79)	24 (31)	<0.001
SIGLEC-1	3,364 (2,568)	1,796 (2,961)	0.004
Median in pg/ml (IQR), n (%); Kruskal-Wallis rank sum test; Fisher´s exact test			

Supplementary Table 5. Absent kidney involvement vs. kidney involvement [minimal time difference to creatinine max], medians and p-values of Wilcoxon rank-sum test. Significant differences (p-values < 0.05) are highlighted in grey.

	No Kidney involvement (n = 132)	Kidney involvement (n = 90)	p-value
s4-1BB	90 (158)	70 (105)	0.084
sCD86	297 (383)	386 (410)	0.214
sCD25	1,332 (1,366)	1,756 (1,927)	0.003
sCTLA-4	2 (9)	0 (7)	0.378
sGal-9	66,188 (91,431)	89,505 (101,063)	0.004
sLAG-3	1,712 (2,834)	1,822 (3,080)	0.695
sPD-1	41 (61)	18 (57)	<0.001
sPD-L1	76 (249)	118 (239)	0.348
sTim-3	11,679 (15,344)	17,301 (21,963)	<0.001
IFN-α2	0 (2)	0 (2)	0.854
IFN-λ2/3	0 (13)	0 (28)	0.059
IFN-λ1	16 (71)	24 (80)	0.636
sCD27	20,115 (21,380)	26,252 (31,932)	0.139
sAPRIL	31,151 (36,538)	23,673 (42,146)	0.084
BAFF	643 (1,496)	852 (1,797)	0.242
IFN-ß	27 (61)	13 (58)	0.238
sPD-L2	8,615 (4,694)	8,265 (6,021)	0.926
sCD40L	28,675 (34,551)	24,694 (30,509)	0.099

Median in pg/ml (IQR)

Supplementary Table 6. Absent anemia vs. anemia [all values], medians and p-value of Wilcoxon rank-sum test. Significant differences ($p < 0.05$) are highlighted in grey.

	No Anemia (n = 245)	Anemia (n = 134)	p-value
s4-1BB	71 (132)	94 (143)	0.023
sCD86	301 (377)	348 (416)	0.219
sCD25	1,233 (1,096)	1,929 (2,084)	<0.001
sCTLA-4	0 (10)	3 (11)	0.014
sGal-9	62,163 (69,251)	108,680 (134,175)	<0.001
sLAG-3	1,586 (2,900)	1,826 (3,191)	0.237
sPD-1	34 (56)	41 (65)	0.332
sPD-L1	74 (203)	150 (243)	<0.001
sTim-3	12,031 (12,201)	22,131 (32,711)	<0.001
IFN-α2	0 (2)	0 (3)	0.023
IFN-λ2/3	0 (21)	0 (22)	0.901
IFN-λ1	23 (75)	34 (79)	0.216
sCD27	20,155 (21,882)	29,863 (30,688)	<0.001
sAPRIL	37,078 (42,158)	35,997 (40,262)	0.864
BAFF	640 (1,309)	1,136 (1,766)	<0.001
IFN-ß	23 (72)	21 (58)	0.322
sPD-L2	8,680 (5,073)	8,328 (5,774)	0.445
sCD40L	28,952 (27,821)	23,843 (26,754)	0.065

Median in pg/ml (IQR)

Supplementary Table 7. Absence of skin involvement vs. skin involvement [all values] medians and p-value of Wilcoxon rank-sum test. Significant differences ($p < 0.05$) are highlighted in grey.

	**No Skin involvement (n = 294)**	**Skin involvement (n = 44)**	**p-value**
s4-1BB	81 (136)	93 (160)	0.732
sCD86	323 (373)	245 (381)	0.634
sCD25	1,428 (1,427)	1,583 (2,187)	0.377
sCTLA-4	0 (10)	0 (14)	0.885
sGal-9	76,268 (95,633)	88,226 (106,360)	0.148
sLAG-3	1,643 (3,191)	1,417 (2,597)	0.290
sPD-1	34 (63)	46 (61)	0.282
sPD-L1	109 (230)	87 (208)	0.703
sTim-3	13,728 (19,700)	13,925 (14,553)	0.784
IFN-α2	0 (3)	0 (2)	0.746
IFN-λ2/3	0 (21)	4 (31)	0.037
IFN-λ1	30 (78)	33 (79)	0.95
sCD27	23,456 (28,709)	22,201 (30,523)	0.760
sAPRIL	35,997 (39,076)	41,661 (36,625)	0.651
BAFF	757 (1,238)	1,394 (3,212)	0.206
IFN-ß	23 (68)	32 (63)	0.592
sPD-L2	8,518 (5,431)	9,423 (5,260)	0.385
sCD40L	27,173 (29,342)	35,791 (29,617)	0.247

Median in pg/ml (IQR)

Supplementary Table 8. Circulating levels of IFN-λ2/3 and IL-2 are not higher in patients with skin involvement compared to patients without skin involvement. Multivariable logistic regression model adjusted for age, sex, and prednisolone dose. Non-significant results (p>0.05) are highlighted in orange.

	**OR**	**p-value**
**IFN-λ2/3**	**1.03**	**0.9**
Age	0.99	0.7
Sex		
f		
m	0.81	0.7
Prednisolone	1	>0.9

Supplementary Table 9. Absent articular involvement vs. articular involvement [all timepoints] medians and p-values of Wilcoxon rank-sum test

	No articular involvement (n = 264)	Articular involvement (n = 30)	p-value
s4-1BB	80 (136)	93 (162)	0.768
sCD86	315 (400)	306 (333)	0.388
sCD25	1,512 (1,689)	1,498 (1,451)	0.606
sCTLA-4	0 (11)	3 (13)	0.348
sGal-9	78,683 (96,526)	82,082 (112,856)	0.240
sLAG-3	1,566 (3,305)	1,927 (2,684)	0.858
sPD-1	35 (65)	30 (52)	0.836
sPD-L1	104 (227)	125 (329)	0.685
sTim-3	13,928 (17,443)	14,793 (16,155)	0.412
IFN-α2	0 (3)	0 (3)	0.889
IFN-λ2/3	0 (22)	4 (29)	0.213
IFN-λ1	30 (79)	23 (74)	0.477
sCD27	22,996 (26,040)	26,465 (45,584)	0.177
sAPRIL	37,329 (40,193)	46,104 (51,954)	0.891
BAFF	858 (1,624)	1,089 (1,949)	0.779
IFN-ß	28 (67)	21 (68)	0.550
sPD-L2	8,725 (5,359)	9,653 (6,103)	0.908
sCD40L	28,129 (28,313)	24,459 (43,642)	0.521

Median in pg/ml (IQR)

Supplementary Table 10. Absent APS findings vs. APS [first timepoints] medians and p-value of Wilcoxon rank-sum test. Significant p-values ($p < 0.05$) are highlighted in grey.

	**No APS (n = 180)**	**APS (n = 54)**	**p-value**
s4-1BB	86 (140)	70 (91)	0.777
sCD86	395 (488)	367 (571)	0.085
sCD25	1,534 (1,685)	1,626 (2,155)	0.510
sCTLA-4	2 (10)	2 (10)	0.961
sGal-9	88,870 (113,516)	100,805 (93,972)	0.176
sLAG-3	1,729 (3,255)	2,024 (3,554)	0.149
sPD-1	35 (62)	30 (91)	0.838
sPD-L1	129 (255)	66 (292)	0.096
sTim-3	15,507 (17,259)	14,940 (16,416)	0.405
IFN-α2	0 (3)	0 (3)	0.856
IFN-λ2/3	0 (23)	0 (25)	0.963
IFN-λ1	30 (80)	35 (69)	0.853
sCD27	23,084 (26,030)	20,192 (28,162)	0.544
sAPRIL	26,411 (35,546)	28,772 (43,200)	0.666
BAFF	728 (1,309)	816 (1,126)	0.807
IFN-ß	14 (62)	31 (69)	0.601
sPD-L2	8,397 (5,399)	7,978 (8,024)	0.747
sCD40L	27,231 (32,264)	20,604 (26,722)	0.134

Median in pg/ml (IQR)

Supplementary Table 11. Comparison between patients in DORIS remission and those not in DORIS remission [first timepoint] (including patients in LLDAS but not in DORIS remission) (medians and p-value of Wilcoxon rank-sum test are listed). Significant p-values ($p < 0.05$) are highlighted in grey.

	DORIS (n = 60)	Non-DORIS (n = 175)	p-value
s4-1BB	81 (126)	74 (132)	0.879
sCD86	388 (507)	381 (457)	0.681
sCD25	1,055 (1,204)	1,721 (1,763)	0.003
sCTLA-4	3 (11)	0.7(9)	0.223
sGal-9	94,366 (103,710)	90,758 (113,164)	0.638
sLAG-3	1,677 (3,042)	1,912 (3,471)	0.458
sPD-1	29 (66)	35 (64)	0.569
sPD-L1	95 (209)	109 (301)	0.294
sTim-3	12,007 (10,435)	16,199 (18,651)	0.046
IFN-α2	0 (2)	0 (3)	0.551
IFN-λ2/3	0 (19)	0 (25)	0.428
IFN-λ1	29 (78)	32 (80)	0.602
sCD27	18,394 (26,611)	23,664 (26,486)	0.361
sAPRIL	24,437 (41,671)	27,513 (35,710)	0.672
BAFF	678 (1,078)	784 (1,455)	0.263
IFN-ß	11 (54)	21 (67)	0.201
sPD-L2	8,034 (6,213)	8,326(5,813)	0.342
sCD40L	23,843 (31,485)	25,478 (31,225)	0.768

Median in pg/ml (IQR)

Supplementary Table 12. Circulating levels of sCD25 and sTim3 are lower in patients in DORIS Remission compared to those not in DORIS remission.
Multivariable logistic regression model adjusted for age, sex, and prednisolone dose.
Non-significant results (p>0.05) are highlighted in orange.

	**OR**	**p-value**
**sCD25**	**1.39**	**0.1**
Age	0.99	0.4
Sex		
f		
m	1.59	0.5
Prednisolone	1.16	0.006

	**OR**	**p-value**
**sTim-3**	**1.30**	**0.2**
Age	0.99	0.4
Sex		
f		
m	1.71	0.4
Prednisolone	1.15	0.007

Supplementary Table 13. DORIS Remission versus neither DORIS nor LLDAS Remission [first timepoint], medians and p-value of Wilcoxon rank-sum test. Significant p-values (p < 0.05) are highlighted in grey.

	DORIS (n = 60)	Neither DORIS nor LLDAS (n = 137)	p-value
s4-1BB	81 (126)	74 (126)	0.811
sCD86	388 (507)	382 (496)	0.745
sCD25	1,055 (1,204)	1,788 (1,860)	0.001
sCTLA-4	3 (11)	2 (13)	0.560
sGal-9	94,366 (103,710)	98,327 (130,274)	0.323
sLAG-3	1,677 (3,042)	1,828 (3,134)	0.596
sPD-1	29 (66)	32 (64)	0.841
sPD-L1	95 (209)	108 (300)	0.277
sTim-3	12,007 (10,435)	16,962 (20,637)	0.016
IFN-α2	0 (2)	0 (3)	0.481
IFN-λ2/3	0 (19)	0 (24)	0.478
IFN-λ1	29 (78)	32 (80)	0.637
sCD27	18,394 (26.611)	24,685 (31,839)	0.223
sAPRIL	24,437 (41,671)	26,944 (38,393)	0.771
BAFF	678 (1,078)	731 (1,693)	0.376
IFN-ß	11 (54)	21 (65)	0.217
sPD-L2	8,034 (6,213)	8,653 (6,107)	0.295
sCD40L	23,843 (31,485)	26,014 (31,644)	0.699

Median in pg/ml (IQR)

Supplementary Table 14. LLDAS Remission versus non-LLDAS Remission [first timepoint], medians and p-value of Wilcoxon rank-sum test. Significant p-values (p < 0.05) are highlighted in grey.

	**LLDAS (n = 98)**	**Non-LLDAS (n = 137)**	**p-value**
s4-1BB	87 (144)	74 (126)	0.753
sCD86	374 (459)	382 (496)	0.869
sCD25	1,315 (1,150)	1,788 (1,860)	0.002
sCTLA-4	2 (8)	2 (13)	0.468
sGal-9	90,345 (86,413)	98,327 (130,274)	0.094
sLAG-3	1,757 (3,359)	1,828 (3,134)	0.920
sPD-1	35 (59)	32 (64)	0.591
sPD-L1	98 (243)	108 (300)	0.391
sTim-3	12,587 (11,455)	16,962 (20,637)	0.0045
IFN-α2	0 (2)	0 (4)	0.429
IFN-λ2/3	0 (23)	0 (24)	0.723
IFN-λ1	30 (79)	32 (80)	0.785
sCD27	19,245 (22,542)	24,685 (31,839)	0.099
sAPRIL	25,835 (38,233)	26,944 (38,392)	0.963
BAFF	768 (1,065)	731 (1,693)	0.770
IFN-ß	13 (58)	20 (65)	0.376
sPD-L2	7,955 (5,255)	8,653 (6,107)	0.320
sCD40L	23,579 (28,326)	26,014 (31,644)	0.678

Median in pg/ml (IQR)

Supplementary Figure 1. ROC curve displaying the predictive performance of univariate logistic regression models for sPD-1 (brown), in differentiating patients with kidney involvement and no kidney involvement.

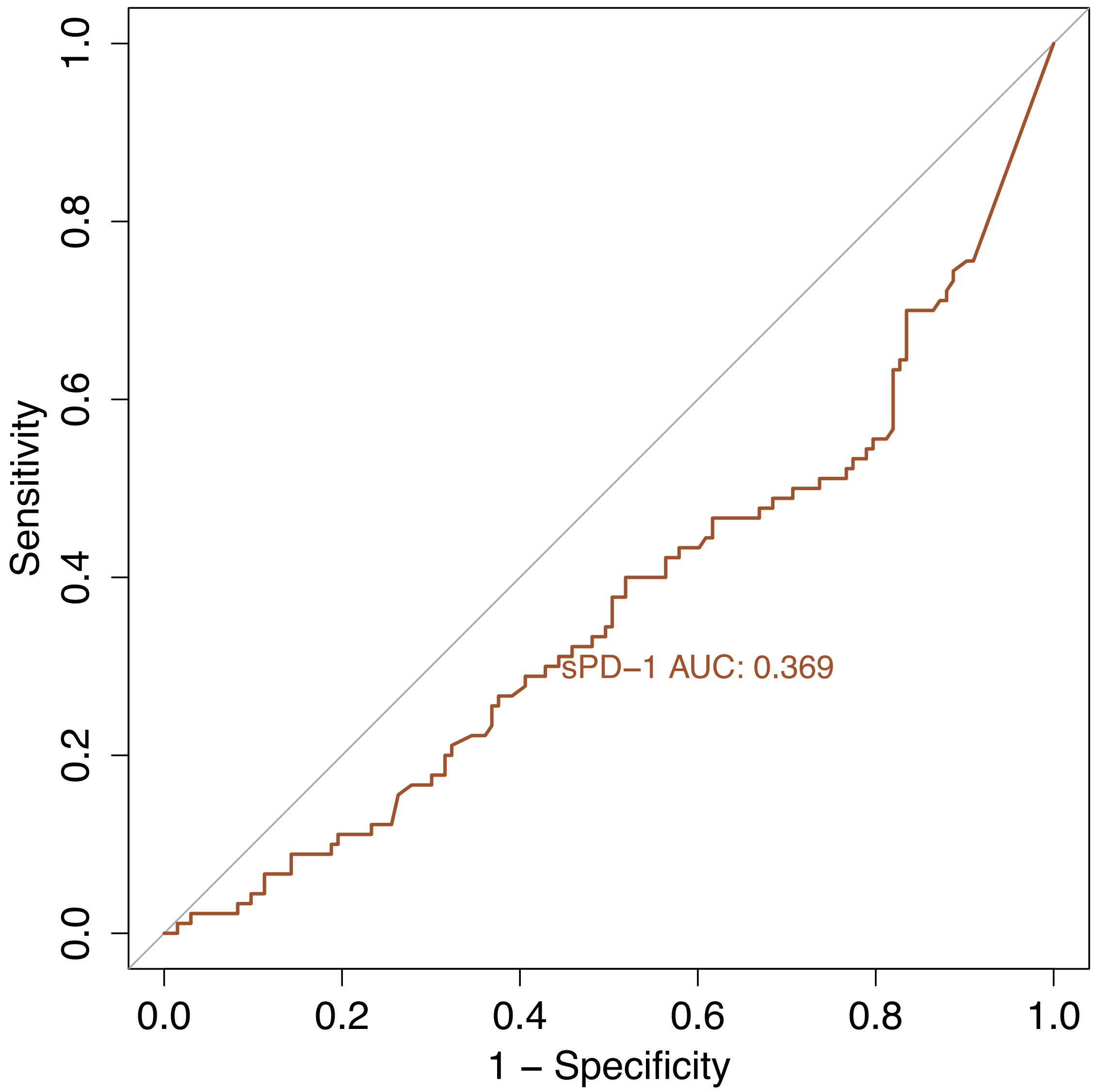

Supplementary Figure 2. Spearman Correlation Matrix [active], samples with SLEDAI > 4) Correlation matrix displaying associations between biomarkers (sCD25, sTim-3 and sGal-9 and BAFF) and other parameters (anti-dsDNA, SIGLEC-1, C3, C4 Proteinuria, Hb, SLEDAI and cSLEDAI). Correlation coefficients are represented by color intensity, with blue indicating positive and red negative correlations.

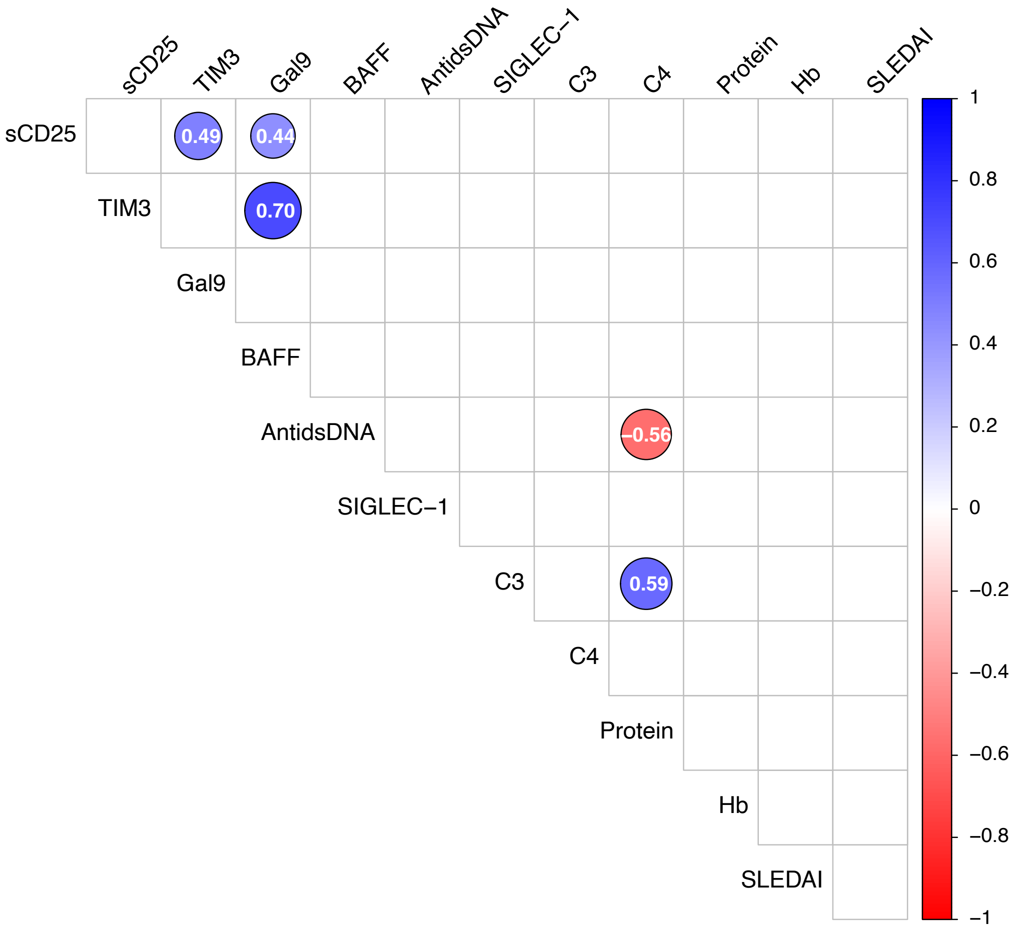

Supplementary Figure 3. Correlations between leukocyte counts and soluble checkpoint molecules
Scatter plots showing correlations between sCD25, sTim-3 and sGal-9 and leukocyte count. Pearson correlation coefficients (r) and p-values are provided for each comparison.

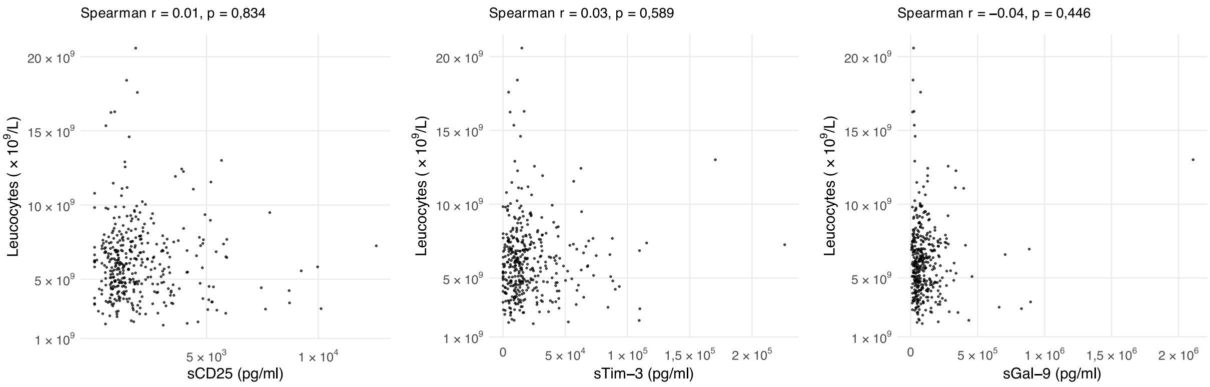

Supplement: Supplementary file 1 [file DataSheet1.pdf]
